# Neither COVID-19, nor cryopreservation, prevented allogeneic product infusion: A report from the National Marrow Donor Program

**DOI:** 10.3389/fimmu.2022.937900

**Published:** 2022-09-20

**Authors:** Nosha Farhadfar, Jeni Newman, Jennifer Novakovich, Jacklyn Barten, Eric T. Ndifon, Jason Oakes, Meghann Cody, Huy P. Pham, Jeffery J. Auletta, John P. Miller, Steven M. Devine, Heather E. Stefanski

**Affiliations:** ^1^ University of Florida, Gainesville, FL, United States; ^2^ National Marrow Donor Program/Be the Match, Minneapolis, MN, United States; ^3^ Center for International Blood and Marrow Transplant Research, National Marrow Donor Program/Be the Match, Minneapolis, MN, United States; ^4^ Nationwide Children’s Hospital, Columbus, OH, United States

**Keywords:** COVID-19, donor registry, unrelated donor, hematopoietic stem cells, National Marrow Donor Program, SARS-CoV-2

## Abstract

**Background:**

The Coronavirus Disease 2019 (COVID-19) pandemic in early 2020 has resulted in an unprecedented level of uncertainty and challenge for the stem cell donor registries. To address these challenges, rapid strategies were implemented by the National Marrow Donor Registry (NMDP) and its network partners. Herein, we aim to report the impact of the COVID-19 pandemic on the collection, utilization of grafts, and short-term outcomes of patients who received stem cell products from COVID-19-positive donors.

**Methods:**

NMDP data during the early phase (1 March 2020 through 1 May 2020) of the pandemic were compared to the later phase (1 March 2021 through 1 May 2021). Odds ratios were calculated to determine the impact of the pandemic on graft sources requested by transplant centers (TCs). The Kruskal–Wallis test was used to test the effect of the pandemic on the disease indication, volume of searches, and number of products not infused.

**Results:**

Although there was an initial decline in overall donor searches during the early phase of the pandemic, these numbers increased reaching pre-pandemic levels during the later phase. Urgent malignant diseases remained the most common indication for transplant in 2021. The pandemic necessitated cryopreservation of stem cell products due to transportation restrictions as well as clinical uncertainties in managing the virus. Cryopreserved grafts remained the most common requested grafts throughout the pandemic. In the later phase of the pandemic, the total numbers of requests for fresh grafts increased, mostly due to the increase in requests for fresh bone marrow (BM) grafts. As the pandemic continued, TCs became more accepting of cryopreservation, resulting in a reduction in the number of products not infused. Lastly, no short-term deleterious outcomes were noted among the patients who had stem cell products infused from a SARS-CoV-2-positive donor.

**Conclusion:**

Throughout the pandemic, the NMDP and TCs worked tirelessly to ensure that patients would receive lifesaving grafts when needed. The data reported here, although limited by small numbers, illustrate that transplantation from donors with COVID-19 is feasible and safe.

## Introduction

A recent study by the National Marrow Donor Registry (NMDP) described the impact of the Coronavirus Disease 2019 (COVID-19) pandemic on transplant center (TC) practices as well as donor registry operations during the initial 3 months of the pandemic (March through May 2020) (1). According to the results, TCs made several modifications in practices based on both institutional and transplant society recommendations to maintain safety of their patients. These changes included focusing on transplanting acute hematologic diseases (ALL, AML, MDS) during the early phases of COVID-19, identifying a backup unrelated donor (URD) for each transplant recipient, increasing the utilization of cryopreserved grafts, and increasing the utilization of direct to workup requests, primarily with domestic URDs. These early phase data were limited by the short timeframes and lack of information on donors and patients’ outcomes, including donors infected by severe acute respiratory syndrome coronavirus 2 (SARS-CoV-2).

Herein, we aim to report the impact of the COVID-19 pandemic on the collection and utilization of URDs during the later phase of the pandemic (March through May 2021). In addition, we evaluated the effect of cryopreservation on the infusion of allogeneic products, analyzing all allogeneic products collected from March 2020 through June 2021. Moreover, we described the short-term outcomes of patients who received stem cell products from COVID-19-positive donors.

## Materials and methods

### Data sources

The data including volume of donor searches, disease indications for allogeneic hematopoietic stem cell transplantation (allo-HCT), graft type, and cryopreservation were collected by the NMDP. The NMDP is a non-profit organization founded in 1986 that operates the Be The Match Registry, the world’s largest registry of unrelated adult donors and umbilical cord blood (UCB) units. Since its inception, the NMDP/Be the Match has facilitated more than 100,000 URD HCTs. NMDP/Be The Match is a global transplant network made up of more than 470 leading centers worldwide, including 155 transplant centers in the United States and 38 international transplant centers (2).

All allo-HCT recipient data were generated from the Center for International Blood and Marrow Transplant Research (CIBMTR). The CIBMTR is a research collaborative between the National Marrow Donor Program/Be the Match Registry and the Medical College of Wisconsin. More than 450 centers around the world contribute detailed clinical, pathological, and outcomes data to the CIBMTR on their patients who undergo HCT.

### Statistical analysis

The early phase (1 March 2020 through 1 May 2020) of the pandemic was compared to the latter phase (1 March 2021 through 1 May 2021). Odds ratios were calculated to determine whether the COVID-19 pandemic had an impact on the type of graft sources. Kruskal–Wallis tests were used to test the effect of COVID-19 on the disease indication for allo-HCT, the frequency of preliminary and formal searches, and the number of products not infused. The p-value <0.01 was considered statistically significant due to carrying out of multiple tests, as a lower alpha level reduces the chance of a false positive.

Analysis of existing data initially gathered for operations and statistical analysis were performed by the NMDP. Data collected included donor collection and infusion dates from March 2020 through 29 July 2021. This timeframe was split into two time periods for COVID-19 comparison: comparing the first 5 months (March 20–July 20) of the pandemic to the last 5 months (March 21–July 21). Kruskal–Wallis tests were performed to test the impact COVID-19 had on products infused. All analyses were completed using the R statistical analysis software (version 4.0.2, https://www.r-project.org/2020), and a p value of <0.05 was significant.

Demographic and baseline characteristics were summarized for all the patients who received stem cell products from COVID-positive donors. Kaplan–Meier estimates were used to describe overall survival probability with death from any cause considered an event, with surviving patients censored at time of last follow-up. Additionally cumulative incidence of neutrophil and platelet recovery with death without hematologic recovery as a competing risk were generated with censoring done at the last follow-up. All analyses were conducted using the SAS statistical analysis software, enterprise guide version 7.15.

## Results

The impact of COVID-19 was evaluated by measuring the number of preliminary and formal donor searches submitted from TCs to the NMDP before March to May 2019, during the early phase (March to May 2020) and the later phase (March to May 2021) of the COVID-19 pandemic. Although a significant decline in overall preliminary and formal searches by both domestic and international TCs during the early onset of the pandemic occurred, searches increased significantly reaching pre-pandemic levels later during the COVID-19 pandemic (**Table 1**). For domestic searches, a significant increase in both preliminary (18% increase, p < 0.01) and formal searches (21% increase, p < 0.01) occurred during March to May 2021 compared to March to May 2020. Similarly, international searches during March to May 2021 significantly increased for both preliminary (17%, p < 0.01) and formal searches (24%, p < 0.01) compared with March to May 2020. Together, these data indicate that volumes of domestic and international searches submitted from TCs to NMDP returned to pre-pandemic levels by March to May 2021.

**Table 1:** Donor searches and disease indications for allogeneic hematopoietic stem cell transplantation.

Preliminary and formal searches				
Domestic TC	Late phase of the pandemic March–May 2021	Early phase of the pandemic March–May 2020	% Change(counts) √	Pre-pandemic March –May 2019
Preliminary	3,974	3,359	+18%	3,873
Formal	2,512	2,081	+21%	2,437
**International TC**				
Preliminary	4,361	3,729	+17%	4,366
Formal	1,498	1,204	+24%	1,756
**Disease indications**				
**By formal date**	March–May 2021	March–May 2020	% Change (Overall)	March–May 2019
**Acute indications**	**2,699 (67%)**	**2,193 (67%)**	**+23%**	**2,706 (65%)**
Domestic TC	1,730	1,415	+22%*	1,636
International TC	969	778	+25%†	1,090
**Non-acute indications**	**1,311 (33%)**	**1,092 (33%)**	**+20%**	**1,427 (35%)**
Domestic TC	782	681	+15%†	828
International TC	529	41	+1190%†	619
**Total**	**4,010**	**3,285**	**+22%**	**4,133**
**By collection date**				
**Acute indications**	**1,094 (72%)**	**1,149 (72%)**	**-5%**	**1,090 (68%)**
Domestic TC	967	1,015	-5%†	953
International TC	127	134	-5%†	137
**Non-acute indications**	**433 (28%)**	**442 (28%)**	**-2%**	**510 (32%)**
Domestic TC	364	362	+1%†	421
International TC	69	80	-14%†	89
**Total**	**1,527**	**1,591**	**-4%**	**1,600**

TC, transplant centers.

**√**Counts refer to number of patients. Acute indications included ALL, AML, and MDS. Non-acute indications include all non-malignant diseases (e.g., bone marrow failure syndromes, hemoglobinopathy). All other malignant diseases were not included in the analysis given their significantly lower frequency as disease indications for allogeneic transplant.

*March to May 2020 versus March to May 2021, p <.01.

†March to May 2020 versus March to May 2021, p >.01.

Urgent malignant diseases (ALL, AML, and MDS) remained to have the most common indication for allo-HCT during the pandemic (**Table 1**). Compared with the initial phase of the COVID-19 pandemic, an overall increase (23%) in the number of formal searches for these indications occurred during the March to May 2021 interval. For domestic patients, there was a 22% increase in the submitted formal searches for malignant disease indications during March to May 2021 compared with March to May 2020 (p < 0.01). An increase in international formal searches for these indications during 2021 (25%) was noted but did not reach statistical significance (p > 0.01). Compared with the early phase of COVID-19, an increase in the number of formal searches by both domestic (15%, p > 0.01) and international TCs (1,190%, p > 0.01) for non-urgent disease indications occurred during the later phase of the pandemic, reaching the pre-pandemic era numbers but was not statistically significant.

Despite a significant increase in formal searches in 2021, conversion rates from formal searches to infusion (collection numbers) declined compared to the early months of the pandemic (2020). Specifically, a 5% decline in collections for urgent disease indications and a 2% decline in collections for non-urgent disease indications occurred during 2021 compared with 2020, although these changes were not statistically significant (p > 0.01). Conversion rates from formal searches to infusion in 2021 returned to pre-pandemic era rates (2019).

Regarding workup requests, there was a 6.5% decrease in total domestic and 3.1% decreased in total international donor workup requests in 2021 compared with 2020 (**Table 2**). This was most likely due to a decrease in requesting multiple donors for workup and an increase in availability of donors at workup. Domestic donors have 77% higher odds of being available at workup in 2021 than 2020 (99% CI: 40%, 124%, p < 0.01). In contrast to the increase noted in domestic donor workup requests, total international workup requests decreased in both the early phase of the pandemic (2020) and late phase of the pandemic (2021) compared to the pre-pandemic era (2019). There was no significant difference in availability of international donors at workup between 2020 and 2021 (p = 0.16). This may also reflect the differences in public health policies concerning the prevention of the spread of COVID-19 which may led to an inability of some international registries to facilitate workup requests for a period of time during the pandemic.

**Table 2:** Donor availability at the time of workup and confirmatory typing.

Donor workup				
Domestic donors	March–May 2021	March–May 2020	% Change (counts 2021 vs. 2020)	Pre-pandemic March–May 2019(% total)
**Available**	850	780	+8%*	763
**Unavailable**	274	444	-62%	194
**Cancelled**	275	325	-18%	259
**Open**	50	1	98%	0
**Total orders**	1,449	1,550	-7%	1,216
**% AV**	76%	64%	+12% (overall % changes)	80%
**International donors**				
**Available**	545	545	0%†	672
**Unavailable**	99	121	-22%	99
**Cancelled**	272	318	-17%	341
**Open**	42	2	+95%	0
**Total orders**	958	986	+3%	1,112
**% AV**	85%	82%	+3% (overall % changes)	87%
**Donor confirmatory typing**				
**Domestic donors**	March–May 2021	March–May 2020 (% total)	% Change (counts 2021 vs. 2020)	Pre-pandemic March–May 2019 (% total)
**Available**	4,635	2,683	+42%*	3,953
**Unavailable**	4,492	3,558	21%	3,759
**Cancelled**	482	1,190	-147%	429
**Open**	6	0	100%	1
**Total orders**	9,615	7,431	23%	8,142
**% AV**	51%	43%	+8% (overall % changes)	51%
**International donors**				
**Available**	3,418	2,107	+38%†	3995
**Unavailable**	1,399	1,013	+28%	1,416
**Cancelled**	378	513	-36%	423
**Open**	17	1	+94%	4
**Total orders**	5,212	3,634	+30%	5,908
**% AV**	71%	68%	+3% (overall % changes)	73%

Counts refer to the number of donor WU or donor CT requests. The percent available (% AV) is defined as AV/(AV + UN). Canceled and open cases do not count in % AV.

*March to May 2020 versus March to May 2021, p <.01.

†March to May 2020 versus March to May 2021, p >.01.

Although the availability of donors at confirmatory testing decreased significantly during the early phase of the pandemic (2020), this returned back to the pre-pandemic phase later on in 2021 (**Table 2**). More specifically domestic donors had 27% higher odds of being available at confirmatory testing in 2021 compared to 2020 (99% CI: 26%, 49%). International donors also had 18% higher odds of being available at CT in 2021 compared to 2020 (99% CI: 4%, 34%). The fact that donors were more available in the later phase of the pandemic led to decreased requests for multiple donors at workup.

The number of peripheral blood stem cell (PBSC) requests significantly increased while the numbers of BM and cord graft requests decreased early on during the pandemic (March to May 2020) compared with the pre-pandemic era (March to May 2019) (1). A decline in BM grafts requests early during the COVID-19 pandemic likely reflects the recommendation by the NMDP to reserve marrow requests only for patients with the greatest need for this graft source to accommodate the network’s capabilities as well as the donors’ limitations in travel. The current analysis looking at graft type requests in 2021 revealed a slight decrease in total number of grafts requested by domestic (6% decrease) and a slight increase in total number of grafts requested by international TCs (2% increase) compared with 2020 (**Table 3**). No significant changes in volume of PBSC requested by TC in 2021 were noted when compared with 2020 (domestic TCs p = 0.09, international TCs p = 0.55). Similarly, no significant change in volume of marrow graft requested by TCs in 2021 compared with 2020 (domestic TCs p = 0.94, international TCs p = 1.0) was observed. Prior to the pandemic, approximately 18% of products delivered by the NMDP were bone marrow (BM), 71% were peripheral blood stem cell (PBSC), and 15% were cord blood. When the pandemic hit, BM products decreased to 12% and cord blood decreased to 11% while PBSC requests increased to 77%. From March to May 2021, bone marrow and PBSC both continued to increase, while cord blood continued to decrease to 7% of the products that were delivered by the NMDP.

**Table 3:** Types of graft requested during the COVID-19 pandemic.

Graft requests				
Domestic TC	March–May 2021	March–May 2020	% Change (counts)√	March–May 2019
BM	380	354	+7%†	523
PB	1,768	1,922	-8%†	1,481
Cord	158	166	-5%	242
**Total**	**2,306**	**2,442**	**-6%**	**2,246**
**International TC**				
BM	68	54	+26%†	80
PB	231	234	-1%†	263
Cord	46	51	-9%	26
**Total**	**345**	**339**	**+2%**	**369**

BM, bone marrow; PB, peripheral blood; TC, transplant centers.

√Counts refer to number of products.

†March to May 2020 versus March to May 2021, p >.01.

**Figure 1:**
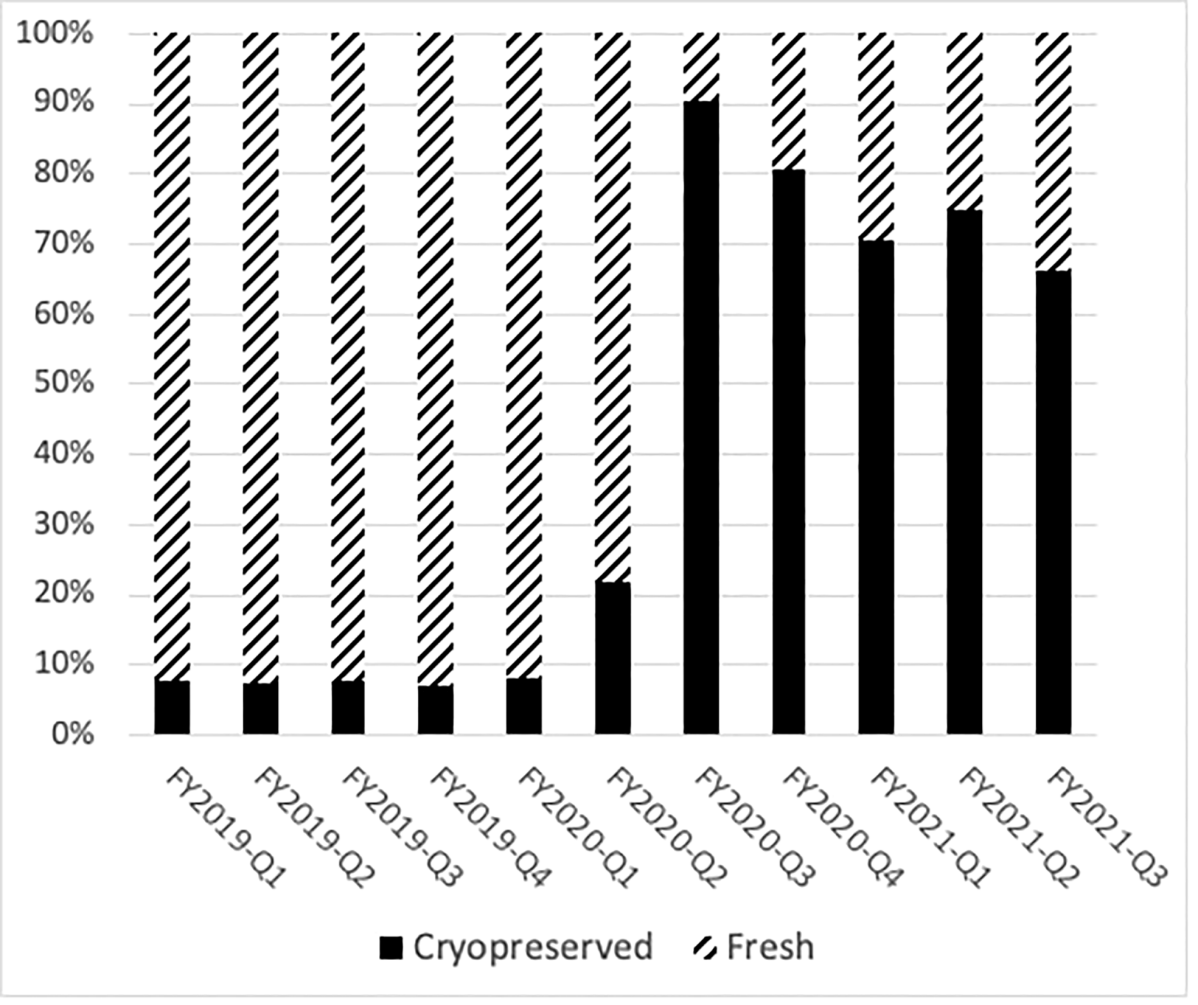
Cryopreservation rates of domestic donor PBSC and marrow collections drastically changed during the pandemic. The percentage of fresh products per quarter is shown in hatched bars, and the number of cryopreserved products is shown in black bars.

Prior to the COVID-19 pandemic, fresh BM or PBSC products were used in more than 90% of transplants. In 2019, for example, 72% of products were fresh PBSC and 19% fresh BM. In the early phases of the COVID-19 pandemic, due to numerous travel restrictions and concerns regarding donor safety, the NMDP temporarily required that the TCs plan for cryopreservation upon receipt of all unrelated and related products facilitated by the NMDP/Be The Match prior to initiation of recipient conditioning (3). The mandate lasted between March and August 2020. In certain clinical circumstances in which fresh product is necessary like bone marrow failure syndromes, identification of a backup graft source was required. Cord blood units were considered as an alternative graft source, as they are already cryopreserved and available. A shift in clinical practice to cryopreserved products during the pandemic led to substantial increase requests for cryopreserved BM and PBSC grafts by both domestic and international TCs during the early phase of the pandemic. In fact, during 2020, the shift to cryopreserved products was seen in both BM (8%) and PB (69%). In the later phase of the pandemic (2021), total numbers of requests for cryopreserved grafts decreased (**Figure 1**), which is mostly due to reduction in TC requests for cryopreserved BM grafts. In 2021, cryopreserved PB remained high at 72% with cryopreserved BM decreasing to 5%. There was a 64% lower odds of requesting cryopreserved BM by domestic TCs (99%, 95% CI 36%–80%) and 83% lower odds of requesting cryopreserved BM by international TCs (99%, 95% CI 12%–97%) in the later phase of pandemic. In contrast to BM grafts, no significant change in rates of requests for cryopreserved PB grafts by domestic (79% in 2020 versus 83% in 2021, p > 0.01) or international (75% in 2020 versus 83% in 2021, p > 0.01) TCs occurred between the early and later phases of the pandemic. As the pandemic continued, there was an increase in fresh BM products and cryopreserved PBSC products domestically in the later phase compared to the early phase (**Table 4**). Internationally, there was an increase in both fresh and cryopreserved BM and an increase in cryopreserved PBSCs from March to May 2021. Interestingly, there were a few centers that had more than 50% fresh products despite the recommendation by the NMDP; most likely this was due to the comfort of the TC infusing fresh products, and it is potentially worrying that the product would not be infused if cryopreserved.

**Table 4:** Graft cryopreservation.

Domestic TC	Graft types	Later phase of the pandemic March–May 2021	Early phase of the pandemic March–May 2020	% Change (counts) √	Prior to pandemic March–May 2019
Not cryopreserved	BM	116	69	68%	229
	PB	189	228	-17%	872
Cryopreserved	BM	63	99	-36%	11
	PB	924	865	+7%†	95
**International TC**					
Not cryopreserved	BM	16	4	+300%	42
	PB	24	40	-40%	172
Cryopreserved	BM	16	2	+700%	4
	PB	118	13	+808%	19

BM, bone marrow; PB, peripheral blood; TC, transplant centers.

√Counts refer to number of products.

†March to May 2020 versus March to May 2021, p >.01.

**Figure 2:**
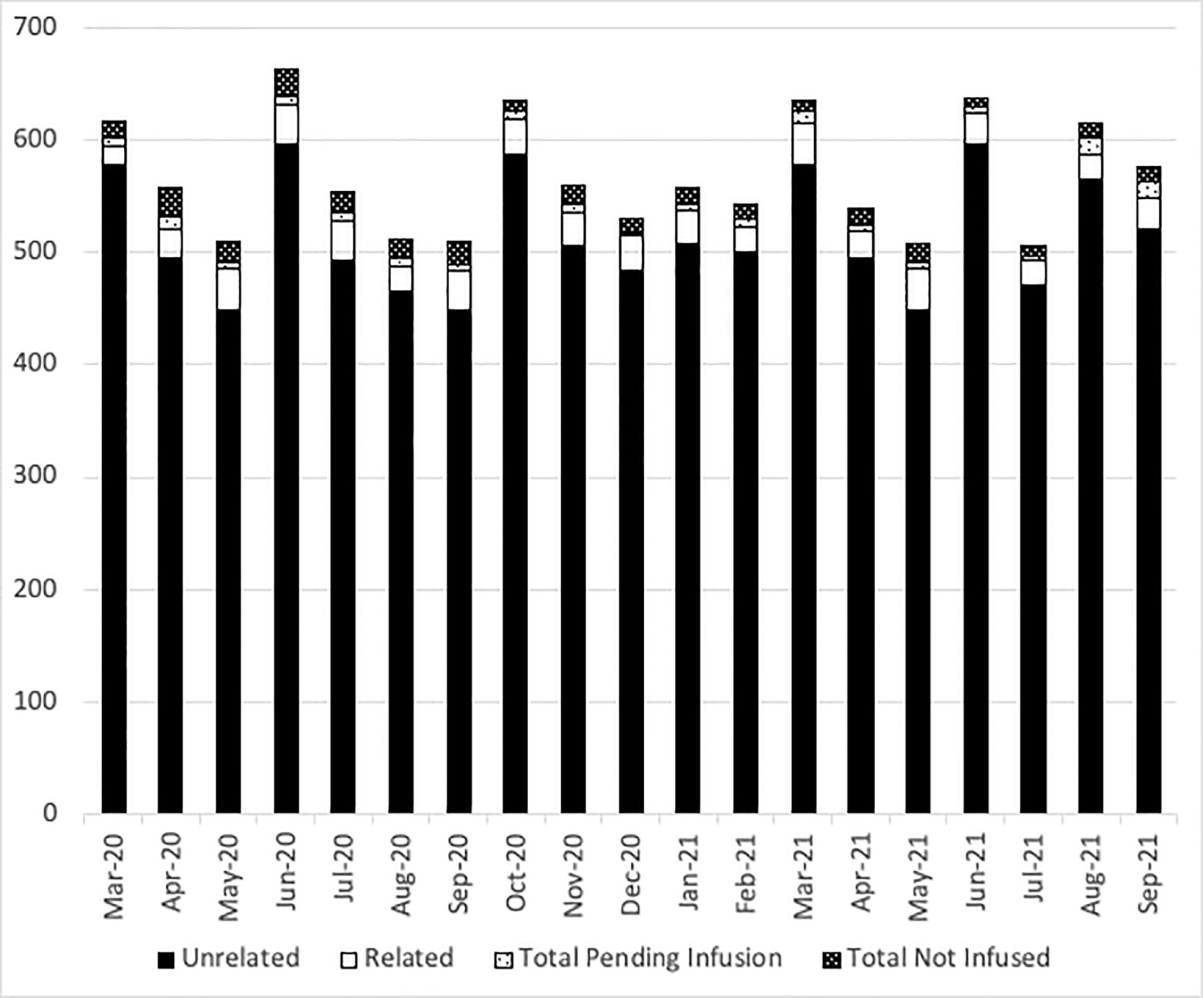
The number of NMDP products delivered each month. Black bars show unrelated donor products, open bars show related donor products, light dots show the number of products pending infusion, and dark dots show the number of products not infused each month from March 2020 to September 2021.

**Figure 3:**
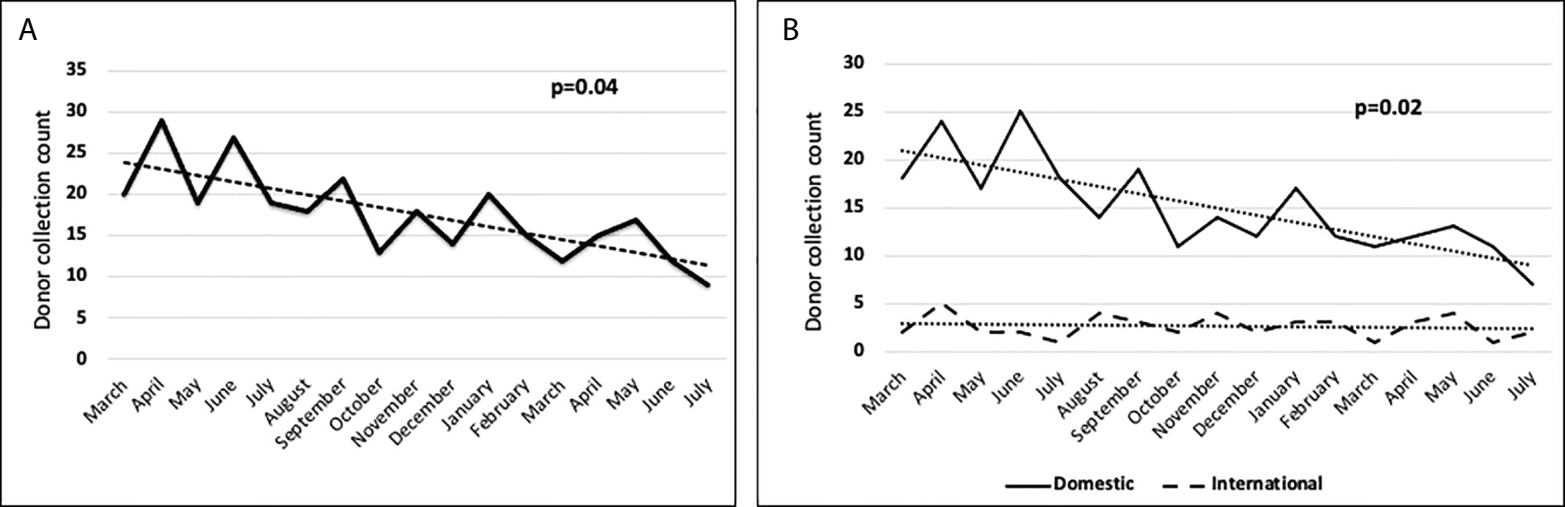
**(A)**The number of stem cell products not infused decreased significantly in the first quarter (March 2020 to July 2020) versus the later quarter of the pandemic (March 2021 to July 2021). **(B)** Decline in the number of products not infused was most pronounced in domestic transplant centers. Data collected included donor collection and infusion dates from March 2020 through 29 July 2021. This timeframe was split into two time periods for COVID-19 comparison, comparing the first 5 months (March 20–July20) of the pandemic to the last 5 months (March 21–July 21). Kruskal–Wallis tests were performed to test the impact COVID-19 had on products infused. p < 0.05 is significant.

**Figure 4:**
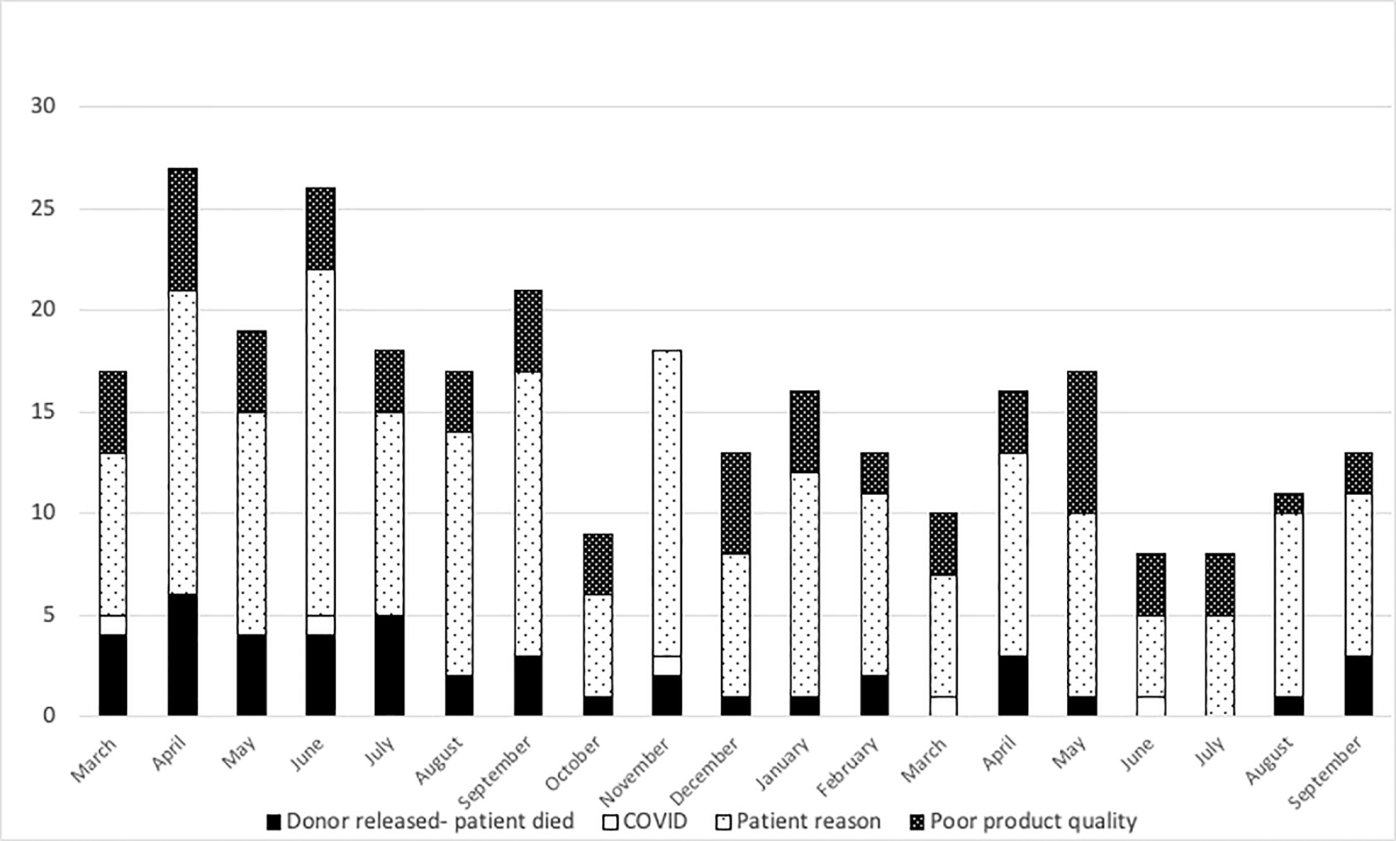
Reasons related to the patient were the most common reasons for the product to not be infused. Black bars show patient death, open bars show COVID-related reasons, light dots show patient reasons, and dark dots show poor product quality from March 2020 to September 2021.

At the NMDP, less than 1% of allograft products were not infused prior to the pandemic. Once the pandemic started and cryopreservation was mandated, the concern arose for the possibility that increased numbers of products would not be infused. Therefore, we assessed whether such an increase occurred within the first year of the COVID-19 pandemic. From 17 March 2020 through 30 September 2021, a total of 10,759 products were collected from related (RD) and unrelated donors (URD). Of these, 10,329 products (96%) were infused at domestic and international TCs (550 were RD and 9,779 were URD allografts). Only 2.7% (289 products) were not infused for a variety of reasons including patient death, patient choice, and poor product quality. Approximately, 1.3% (141 products) are pending infusion at the time of this manuscript (**Figure 2**). The products that were not infused were predominantly URD products. We hypothesized that as TCs became more adept at cryopreservation, the number of uninfused products would decrease. To evaluate this hypothesis, we compared the number of grafts infused in the first quarter (March 2020 to July 2020) versus the later quarter of the pandemic (March 2021 to July 2021). In support and as shown in **Figures 3A** and **3B**, the number of products not infused decreased significantly (p = 0.04). Of note, a decline in the percent of products not infused was most pronounced in domestic TCs (p = 0.02). Small sample sizes precluded analysis for international TCs. Reasons for a product not being infused were mostly related to recipient medical conditions including infection, relapse of the primary disease, and worsening or new comorbidities (**Figure 4**). However, poor product quality (viability, contamination, cell dose) was also a reason for not infusing a product. In four circumstances, TCs chose not to infuse the product due to the donor testing positive for SARS-CoV-2 after donation. Such hesitancies to infuse products from SARS-CoV-2^+^ donors led us to investigate both the number of SARS-CoV-2^+^ donors and the outcomes in recipients who received products from SARS-CoV-2^+^ donors reported to the CIBMTR.

A total of 34 cases of SARS-CoV-2^+^ URDs between March 2020 and August 2021 were identified. Among the 34 cases, 13 had donors with COVID-19 symptomatology that caused the collection to be stopped, 17 had a donor that tested positive for SARS-CoV-2 post-donation, and four were uncharacterized. Out of 17 post-donation SARS-CoV-2^+^ donors, 13 products (76%) were infused and four were not infused based on the TC’s preference. Donors were requested to contact the NMDP if they tested positive for SARS-CoV-2 or had symptoms consistent with COVID-19 within 30 days after donation. Of the 13 products infused, nine (69%) were from donors that tested positive for SARS-CoV-2 or had COVID-19 symptoms within 7 days of donation. The demographics of the patients who received stem cell products from SARS-CoV-2+ donors and had reported outcomes are listed in **Table 5**. Most patients were older than 60 years, were Caucasian, and had acute myeloid leukemia. The median follow-up was approximately 6 months. Among those receiving products from SARS-CoV-2+ donors, no recipients subsequently developing COVID-19 were reported. Moreover, no deleterious effects on neutrophil and platelet engraftment were observed, and 6-month overall survival was 100% (**Table 6**).

**Table 5:** Characteristics of hematopoietic stem cell transplant (HCT) recipients from donors that tested positive for COVID-19 post-donation.

Characteristic	N (%)
No. of patients	7
**Age at HCT—no. (%)**	
30-39	1 (14)
50-59	1 (14)
>60	5 (71)
**Sex—no. (%)**	
Male	3 (43)
Female	4 (57)
**Ethnicity—no. (%)**	
Non-Hispanic or Latino	6 (86)
Not reported	1 (14)
**Race—no. (%)**	
White	6 (86)
Not reported	1 (14)
**Disease—no. (%)**	
AML	5 (71)
MDS	1 (14)
ALL	1 (14)
**Follow-up—median (range)**	6 (6-13)

**Table 6:** Outcomes of patients who received stem cell products from COVID-19 positive donors.

Outcomes	N	Prob (95% CI)
**Overall survival**	7	
3 months		100%
6 months		100%
**Neutrophil recovery**	7	
14 days		14.3 (0-50.2) %
28 days		100%
45 days		100%
**Platelet recovery**	6	
14 days		0%
28 days		66.7 (21.6-98.0) %
45 days		83.3 (33.8-100) %
60 days		100%

## Discussion

As COVID-19 significantly impacted healthcare systems, data herein show that transplant centers made practice adjustments to safely provide their patients with potentially life-saving cellular therapies, including allogeneic HCT. Similarly, the NMDP was rapidly adapted in continuing to provide safe and essential donor registry services. As a reflection, volumes for preliminary searches, numbers of infused grafts, and types of grafts requested did not substantially differ between later times in the pandemic and pre-pandemic. Lastly, although some TCs have been hesitant to infuse products from SARS-CoV-2^+^ donors, our limited data suggest that recipients receiving products from these donors does not adversely affect engraftment or survival (4).

In the early months of the COVID-19 pandemic, there was a significant reduction in the number of both domestic and international searches submitted from TCs, reflecting a reduction in the number of transplants performed due to concerns of infection transmission and resource allocation. Such reduction in allogeneic HCT was due to a decrease in transplant numbers for non-malignant disease indications as recommended by professional societies like the ASTCT and EBMT to prioritize transplants. Despite the decline in formal searches in 2020, conversion rates from formal search to infusion remained similar to or higher than before the pandemic. In the later phase of the pandemic (2021), numbers of formal searches increased significantly compared to earlier in pandemic (2020) nearly reaching pre-pandemic numbers (2019). 2021 conversion rates from formal search to infusion also returned to pre-pandemic era rates.

Changes in patterns of graft requests and rates of cryopreservation during the pandemic were observed. Prior to the pandemic, annual rates for cryopreservation ranged from 5% to 8% and were largely due to patient-related complications like acute infection or disease progression. At the onset of the pandemic in March 2020 to ensure patients’ safety, the NMDP made the decision to strongly recommend and then to require cryopreservation of all unrelated and related products (with limited exceptions) prior to initiation of recipient conditioning, leading to a drastic shift toward the use of cryopreserved BM and PBSC grafts (3). This was not adopted at all TCs; most TCs had >75% of their products cryopreserved, but there were a few notable exceptions that preferred fresh products most likely due to comfort with fresh product infusion. NMDP became more adept at facing logistical challenges and was able to accommodate more non-cryopreserved products in August 2020; the number of cryopreserved grafts declined in early 2021 (75% of total grafts). Compared to the early phase, the later phase of the pandemic associated with a significant decrease in the requests for cryopreserved marrow products by TCs. One potential reason for this decrease was a modification in the NMDP cryopreservation requirement for patients with severe aplastic anemia (SAA) and other bone marrow failure diseases in order to receive non-cryopreserved allografts in May 2020, given unfavorable outcomes (graft failure) reported in SAA patients receiving cryopreserved grafts (5). Significant loss of total nucleated cells after cryopreservation of BM graft may have contributed to graft loss in SAA patients (6).

Several challenges attributed to the pandemic could impact allograft infusions. Ideally, BM and PB allograft need should be infused or cryopreserved within 72 h of collection (7). Recent papers have emphasized the importance of transit time. In a recent study at Dana Farber Cancer Institute, researchers looked at outcomes of 101 recipients who received cryopreserved grafts and found that grafts >48 h old at the time of cryopreservation or infusion significantly increased risk of graft failure (hazard ratio = 4.57; 95% confidence interval, 1.71–12.3; p = .0025) (8). However, the travel restrictions may occasionally result in products delivery exceeding the 72-h limit. For this reason, the NMDP employed a hub and spoke model with international donor networks to mitigate transit issues; however, despite these measures, there were times when products were delivered out of these time frames. For products exceeding the 72-h collection, the NMDP recommends processing and cryopreservation according to TC standard protocols. In addition, pre- and post-cryopreservation cell counts and viability are recommended to assess product quality prior to subsequent infusion. One concern that has been brought up by multiple registries is that products that are cryopreserved will not be infused (stem cell donor registry activities during the COVID-19 pandemic: a field report by DKMS and others). In order to determine the impact of the COVID-19 pandemic on infusion of cryopreserved products, we evaluated the percent of products not infused over the course of the pandemic. Prior to the pandemic, <1% of products were not infused. During the pandemic, only 2.7% of products were not infused, and the number of non-infused products drastically reduced as the pandemic continued. DKMS estimated that up to 10% of cryopreserved products were not infused (9, 10), an estimate greater than our estimate of 2.7% not infused with 1.3% of products pending infusion. The difference between registries could be related to multiple factors, including collecting donors closer to the planned transplant, ensuring that collections only occur in patients that are in a deep remission, or patients having less comorbidities preventing them from undergoing transplant. Also, donors were informed about the risk that the product donated may not be infused. Regardless, these data reassure that almost all products are infused despite being cryopreserved.

Lastly, we herein presented 6-month survival rates of seven patients who potentially could have been contaminated with SARS-CoV-2. Based on our study, there were no short-term deleterious HCT outcomes noted among the patients who had stem cell products from a donor who tested positive for SARS-CoV-2. Moreover, transmission of COVID-19 was not seen in any of these patients. A similar finding was reported in a recent survey study evaluating outcomes of the five related donors who tested positive at medical evaluation, start of mobilization, collection, and 18 days after donation (11). The transmission of SARS-CoV-2 was not detected in any of the three patients who received products from COVID-19-positive donors. Data from the remaining two patients were not reported. There are also a few case reports indicating lack of transmission of the virus in patients who were transplanted from donors positive for SARS-CoV-2 (11–13). Currently, there is no evidence that SARS-CoV-2 is transmissible in the blood (14). There is also lack of evidence that respiratory viruses can survive cryopreservation or other pathogen-reduction methods. Although these data are limited by the small numbers and lack of information on the vaccination status of HCT recipients, it nonetheless illustrates that transplantation from COVID-19 donors is feasible and viral transmission through stem cell transplantation is less likely.

The COVID-19 pandemic continues to pose logistical challenges to the NMDP and other global URD registries, even in 2022, particularly during the most recent Omicron subvariant BA.5 surge. While <10% of NMDP products were cryopreserved prior to the pandemic, >90% of products have been cryopreserved during the mandate periods (23 March 2020–10 August 2020 and 17 January 2022–14 March 2022). Rates have also varied by graft source: 68% of PB products and 27% of BM products are currently being cryopreserved (July 2022). There have been increases in donors testing positive between medical clearance and infusion of their product to the recipient since the Omicron surge and donor availability continues to be a challenge. TCs ordering fresh products for infusion following initiation of conditioning should be aware of these issues and have backup donors or stem cell products ready. In summary, throughout the pandemic, the NMDP, a network of TCs, and collection/apheresis centers overcame tremendous obstacles and quickly adapted their processes so patients could continue to receive life-saving products. Despite cryopreservation, a surprisingly low number of products were not infused. Moreover, NMDP search and allograft volumes resumed to pre-pandemic levels later in the pandemic and grafts from SARS-CoV-2+ donors did not result in harmful effects, emphasizing limited data.

## Data availability statement

The original contributions presented in the study are included in the article/supplementary material. Further inquiries can be directed to the corresponding author.

## Ethics statement

Ethical review and approval was not required for the study on human participants in accordance with the local legislation and institutional requirements. Written informed consent for participation was not required for this study in accordance with the national legislation and the institutional requirements.

## Author contributions

HS and SD designed the project, edited and reviewed the final manuscript. NF wrote the manuscript. JNe, JNo, JB and EN did data analysis and provided figures. HS, SD, NF, JO, MC, JM and JA discussed and reviewed the data. All authors contributed to the article and approved the submitted version.

## Conflict of interest

JNe, JNo, JB, EN, JO, MC, HP, JA, JM, SD and HS are employees of the National Marrow Donor Program.

The remaining author declares that the research was conducted in the absence of any commercial or financial relationships that could be construed as a potential conflict of interest.

## Publisher’s note

All claims expressed in this article are solely those of the authors and do not necessarily represent those of their affiliated organizations, or those of the publisher, the editors and the reviewers. Any product that may be evaluated in this article, or claim that may be made by its manufacturer, is not guaranteed or endorsed by the publisher.
